# Camptothecinoids from the seeds of Taiwanese *Nothapodytes foetida*

**DOI:** 10.3390/molecules13061361

**Published:** 2008-06-16

**Authors:** Shou-Fang Wu, Pei-Wen Hsieh, Chin-Chung Wu, Chia-Lin Lee, Shu-Li Chen, Chi-Yu Lu, Tian-Shung Wu, Fang-Rong Chang, Yang-Chang Wu

**Affiliations:** 1 Graduate Institute of Natural Products, Kaohsiung Medical University, Kaohsiung 807, Taiwan; E-mails: u9557006@kmu.edu.tw (S.F. Wu); pewehs@kmu.edu.tw (P.W. Hsieh); yachwu@kmu.edu.tw (Y.C. Wu); 2 Center of Excellence for Environmental Medicine, Kaohsiung Medical University, Kaohsiung 807, Taiwan; 3 Department of Biochemistry, Kaohsiung Medical University, Kaohsiung 807, Taiwan; E-mail: cylu@kmu.edu.tw (C.Y. Lu); 4 Department of Chemistry, National Cheng Kung University, Tainan 701, Taiwan; E-mail: tswu@mail.ncku.edu.tw

**Keywords:** *Nothapodytes foetida*, 9-methoxy-18,19-dehydrocamptothecin, 5-hydroxy-mappicine-20-*O*-β-glucopyranoside, camptothecin, cytotoxicity

## Abstract

Two new alkaloids, 9-methoxy-18,19-dehydrocamptothecin (**1**) and 5-hydroxymappicine-20-*O*-β-glucopyranoside (**2a/2b** as a racemic mixture), together with nine known compounds: camptothecin (**3**), 9-methoxy-camptothecin (**4**), 5-hydroxy-camptothecin (**5a**/**5b** racemic mixture), 5-hydroxy-9-methoxycamptothecin (**6a**/**6b** racemic mixture), diosmetin (**7**), apigenin (**8**), apigenin-7-*O*-glucopyranoside (**9**), rosin (cinnamyl-*O*-β-D-glucopyranoside) (**10**) and amarantholidoside IV (**11**) were isolated from the immature seeds of *Nothapodytes foetida* (Wight) Sleumer. The structures were elucidated by spectroscopic analyses. In the present research, compounds **1**, **3**, **4**, **5a**/**5b** and **6a**/**6b**, also showed *in vitro* cytotoxicity against six cancer cell lines (HepG2, Hep3B, MDA-MB-231, MCF-7, A549, and Ca9-22). Among them, compound **1** exhibited significant cytotoxicity against these cancer cell lines, with IC_50_ of 0.24-6.57 μM. Furthermore, HPLC profiles were developed for qualitative and quantitative analysis of these active constituents in different parts of this plant, including mature and immature seeds, leaves, stems and roots. The results revealed that compounds **3** and **4** have the highest concentrations, which are found in the roots part of the plant.

## Introduction

Camptothecin, a modified monoterpene indole alkaloid, was first isolated from *Camptotheca acuminata* (Joy Tree, Nyssaceae) [1], a tree native to China. Subsequently, camptothecin has been found in other plant species, inculding *Nothapodytes foetida* [2], *Merrilliodendron megacarpum* [3], *Pyrenacantha klaineaqna* (Icacinaceae) [4], *Ophiorrhiza mungos* [5], *Ophiorrhiza pumila*, *Ophiorrhiza filistipula* [6], *Ophiorrhiza trichocarpon* [7] (Rubiaceae), *Ervatamia heyneana* (Apocynaceae) [8], and *Mostuea brunonis* (Loganiaceae) [9]. In 1972, Govindachari *et al.* found that *N. foetida* (formerly, *Mappia foetida* Miers) [2] is a rich source of camptothecin and 9-methoxy-camptothecin [10]. Camptothecin exhibits an anti-tumor action due to its inhibitory activity to DNA topoisomerase I [11]. Two derivatives of camptothecin, irinotecan and topotecan, were developed to improve the water-solubility, and were approved for use against breast cancer by U.S. Food and Drug Administration (FDA) in 1996 [12].

The development of camptothecin-containing plants as a cash crop is becoming an important issue in southeastern Asia. *Camptotheca acuminata* and *Nothapodytes foetida* were both cultured in Taiwan successfully. *N. foetida* is the only species native to Orchid Island, where it is used for hedges or firewood and is cultured in Taiton Hsien, Taiwan [13]. In 1995, we published a paper that revealed a new camptothecinoid from *N. foetida* [14]. Meanwhile, the anti-cancer agent, “*Campto Injection*,” [Irinotecan Hydrochloride] was approved as a medicine for treating several cancers in Japan, France, and United States with camptothecin originating from Taiwanese *N. foetida*.

Since Taiwanese *N. foetida* is an important resource for anticancer drugs, this plant has been reinvestigated. A number of camptothecinoids, other alkaloids and phytochemicals have been reported from this plant [13,14,15]. In the current investigation, camptothecinoids, including two new ones, have been identified and quantified by HPLC, in different parts of Taiwanese *N. foetida*, including mature and immature seeds, leaves, stems, and roots.

The structures of two new camptothecinoids were elucidated by spectroscopic analyses and the cytotoxicity of camptothecinoids toward six cancer cell lines (HepG2, Hep3B, MDA-MB-231, MCF-7, A549, and Ca9-22) was investigated.

## Results and Discussion

**Figure 1:**
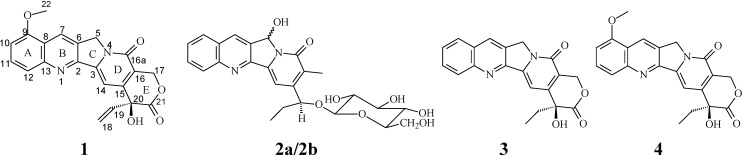
The structures of compounds **1**-**4** from *N. foetida*.

Five camptothecinoids, compounds **1**, **3**, **4**, **5a**/**5b**, and **6a**/**6b** (**5a**/**5b** and **6a**/**6b** are racemic mixtures), were isolated from organic layer extracts of the immature seeds of *N. foetida*. The other new compound, **2**, was isolated from a more polar fraction. The extracts from different parts of Taiwanese *N. foetida* were investigated for compounds **1**, **3**, **4**, **5a**/**5b** and **6a**/**6b** and evaluated for the content of camptothecin (**3**) and 9-methoxy-camptothecin (**4**) (Figure 1).

### Determination of Isolated Compounds

HRESIMS of compound **1** showed an [M+H]^+^ ion at *m/z* 377.1135, corresponding to the molecular formula C_21_H_17_N_2_O_5_. The IR spectrum indicated the presence of hydroxyl (3406 cm^-1^), lactone carbonyl and lactam carbonyl (1745 cm^-1^ and 1652 cm^-1^) and aromatic functional (1500 cm^-1^) groups. By comparing to mass data of 9-methoxy-camptothecin (**4**), the molecular formula reduces two protons of **1**. According to its ^1^H- and ^13^C-NMR spectra, new compound **1** was similar to 9-methoxy-camptothecin (**4**). Compound **1** was found to be a 18,19-dehydro-analog of **4** with the following changes in the NMR chemical shifts: a typing ABC spin coupling pattern attributed to a vinyl group of **1** [δ 5.34 (1H, d, *J=*17.4), 5.33(1H, d, *J=*10.2), and 5.99 (1H, dd, *J*=10.2, 17.4)] instead of an ethyl group of **4** [16]. The HMBC spectrum showed the correlations of H-18/C-19 and H-18/C-20 as well as H-19/C-20, which confirmed the vinyl group was located at C-20. Thus, the new compound **1**, was determined as 9-methoxy-18,19-dehydrocamptothecin.

HRESIMS of the new compound **2** showed an [M+H]^+^ ion at *m/z* 485.1925, corresponding to the molecular formula C_25_H_29_N_2_O_8_. The IR spectrum indicated the presence of hydroxyl (3397 cm^-1^), lactam carbonyl (1670 cm^-1^) and aromatic functional (1596 cm^-1^) groups. In the ^1^H-NMR spectrum of compound **2**, the resonances and multiplicities largely matched those of the A, B, and D rings in camptothecin (**3**), however, in ^1^H- and ^13^C-NMR spectra, the overlapping signals and two sets of signals for part of molecules could be distinguished, revealing **2** to be a racemic mixture. The absence of geminal protons (H-7) at δ 5.43, the absence of lactone carbonyl (C-21) at δ 172.5, and the presence of a singlet at δ 2.25 (3H, H-17) and a triplet at d 5.27 (1H, *J*=6.9 Hz, H-20) suggested that **2** lacked the E ring of camptothecin. As another difference in the ^1^H-NMR spectra, compound **2** showed a pair of downfield-shifted singlets at δ 6.97/7.00 (1H for **2a** and **2b** in a ratio of 1:1) in place of the proton singlet at δ 5.28 (H-5, 2H) found in **3**. In addition, in the ^13^C-NMR spectrum, C-5 was found at δ 84.3/84.4 in **2**, rather than 50.6 as in **3**. Based on this data, a hydroxyl group was attached on C-5. The NMR data for 5-hydroxyl substitution of camptothecinoids could be assigned on the basis of data of the known synthetic product, 5-hydroxycamptothecin [17]. The proton signals between δ 3.18 to 4.10 were assigned to a sugar moiety. The coupling constant of the anomeric proton (δ 4.10, d, *J*=7.8, H-1') indicated the β-configuration, and H-1’ showed a HMBC correlation with C-20. This established that the glucose residue was attached at C-20. For the C-20, it could not be found clear positive or negative peak between 300-400 nm. We defined C-20 in an *S* configuration according to the past literature [18]. Because of the limited amount of **2**, we could not determine the stereochemistry of C-5 in means of chemical reactions, such as the Mosher ester method. Therefore, the stereochemistry of C-5 remains undefined and the structure of compound **2**, was determined as 5-hydroxy-mappicine-20-*O*-β-glucopyranoside.

**Table 1:** ^1^H-NMR (600 MHz) and ^13^C-NMR (125 MHz) spectral data of compounds **1 **(in DMSO) and **2** (in CD_3_OD) (δ in ppm, *J* in Hz).

	^1^H-NMR		^13^C-NMR	
Position	**1**	**2a/2b**	**1**	**2a/2b**
1				
2			152.6	152.9/153.0
3			146.3	141.9
4				
5	5.28 (2H, s)	6.99/7.00 (1H, s)	50.6	84.3/84.4
6			129.2	133.1
7	8.87 (1H, s)	8.58/8.59 (1H, s)	126.1	134.5
8			120.0	129.9
9		8.06 (1H, d, 7.8),	154.9	130.1
10	7.18 (1H, d, 7.8)	7.85 (1H, t, 7.8)	106.0	132.2
11	7.78 (1H, dd, 7.8, 8.4)	7.67 (1H, t, 7.8)	130.6	128.8
12	7.73 (1H, d, 8.4)	8.12 (1H, d, 7.8)	121.1	129.7
13			148.8	150.4
14	7.32 (1H, s)	7.59 (1H, s)	96.8	101.8/101.9
15			148.5	153.2/153.3
16			119.4	130.6/130.7
16a			156.7	163.5
17	5.37 (2H, dd, 15.6, 16.2)	2.25 (3H, s)	65.1	12.2
18	5.33 (1H, d, 10.2)	1.00 (3H, t)	117.1	10.1
	5.34 (1H, d, 17.4)			
19	5.99 (1H, dd, 10.2, 17.4)	1.89 (2H, m)	134.2	29.9
20		5.27 (1H, t)	73.4	76.6
21			170.8	
-Ome	4.05 (3H, s)		56.2	
-OH	7.06 (1H, s)			
20-*O-*β*-*glucoside				
1'		4.09/4.10 (1H, d, 7.8)		101.7
2'		3.21- 3.35 (1H, m)		75.2
3'		3.21- 3.35 (1H, m)		77.9
4'		3.21- 3.35 (1H, m)		71.8
5'		3.18 (1H, m)		78.2
6'a		3.91 (1H, m)		62.9
6'b		3.69 (1H, m)		

### Qualitative and quantitative analysis

Compounds **1** (1.69 mg), **3** (11.51mg), **4** (3.79 mg), **5a**, **5b** (8.19 mg), and **6a**, **6b** (8.12 mg) were obtained from the organic layer. Separation on a reversed phase C-18 column (250×4.6 mm) with acetonitrile-H_2_O (25:75, v/v) as a solvent system provided good separation of these camptothecinoids. The HPLC profile of a mixture of compounds **1**, **3**, **4**, **5a**/**5b** and **6a**/**6b** carried out by the above condition is shown in Figure 2A. The crude extracts from different parts of *N. foetida* were prepared and analyzed, and the HPLC profiles were shown in Figure 2B.

**Figure 2A:**
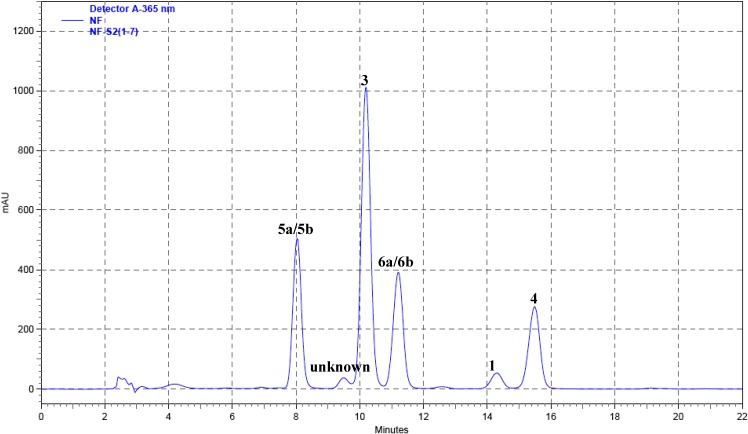
Qualitative HPLC profile of a mixture of compounds **1**, **3**, **4**, **5a**/**5b** and **6a**/**6b** identified in *N. foetida*.

**Figure 2B:**
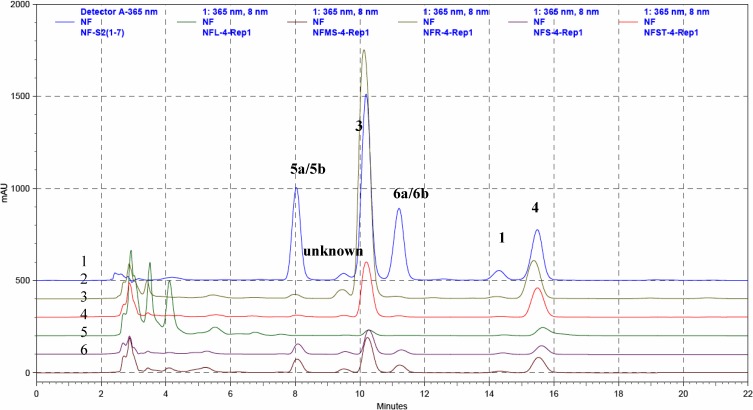
The HPLC profiles of different parts of crude extracts from the top to bottom are: 1. mixture of compounds **1**, **3**, **4**, **5a**/**5b** and **6a**/**6b**; 2. roots; 3. stems; 4. leaves; 5. immature seeds; and 6. mature seeds.

Calibration curves were established with six concentrations (0.015-0.5 mg/mL) of compounds **3** and **4** (see Experimental section). The linearity of the plot of concentration (x, mg/mL) for each compound versus peak area (y) was investigated. Under these analytical conditions, good linearities for all of the calibration curves were obtained (Table 2).

**Table 2:** Regression equations and retention times of compounds **3**, **4** determined for the HPLC assay.

Compound	Rt (min)	Regression equation	Linear range (mg/mL)	R^2^
Camptothecin (**3**)	10.186	y = 4E+07x + 138346	0.015625-0.5	0.9998
9-methoxy-camptothecin (**4**)	15.478	y = 3E+07x + 134716	0.015625-0.5	0.9999

As indicated in Table 3, camptothecin (**3**) was more abundant in the roots (9.73%) than other parts of *N. foetida*. The other important component, 9-methoxy-camptothecin (**4**), also showed the highest concentration in the roots.

**Table 3:** The contents of compounds **3**, **4** from different parts of *N. foetida* (g/Kg).

Plant part	Leaves	Mature seeds	Roots	Immature seeds	Stems
Camptothecin (**3**)	0.58	0.54	15.59	0.40	1.78
9-methoxy-camptothecin (**4**)	1.80	0.38	3.85	0.21	1.54

### Cytotoxicity of Isolated Compounds

Compounds **1**-**4**, **5a**/**5b** and **6a**/**6b** were screened in an *in vitro* cytotoxicity assay. Compounds **1**, **3**, **4**, **5a**/**5b** and **6a**/**6b**, but not compound **2**, showed cytotoxic activity against HepG2, Hep3B (human liver cancer), A549 (human lung carcinoma), MDA-MB-231, MCF-7 (breast carcinomas) and Ca9-22 (human oral squamous carcinoma). Doxorubicin was used as a positive control, and the data shown in Table 3. In this report, **1** showed a significant cytotoxicity against the six cancer cell lines, especially towards Ca9-22 with IC_50_ of 0.24 μM. Additionally, compound **1** exhibited the higher cytotoxicity against HepG2 (IC_50_ 3.43 μM) than **3**, **4**, **5a**/**5b**, and **6a**/**6b** (IC_50_ 39.52-42.06 μM).

**Table 3:** Cytotoxicity of compounds **1**-**4**, **5a**/**5b** and **6a**/**6b** against six cancer lines (IC_50_: μM).

Compound	HepG2	Hep3B	MDA-MB-231	MCF-7	A549	Ca9-22
**1**	3.43	3.80	6.57	6.22	2.77	0.24
**2a**/**2b**	-	-	-	-	-	-
**3**	44.02	0.40	2.36	0.37	0.11	0.02
**4**	41.01	0.58	1.88	0.37	0.16	0.01
**5a**/**5b**	42.06	8.10	-	35.25	5.44	8.11
**6a**/**6b**	39.52	2.87	26.78	12.39	5.20	1.85
**Doxorubicin**	0.15	0.33	0.28	0.18	0.24	0.22

“-”: cytotoxicity > 20 μg/mL

## Experimental

### General

Optical rotations were recorded on a JASCO P-1020 polarimeter. IR spectra were measured on a Perkin Elmer system 2000 FT-IR spectrophotometer in CHCl_3_. UV spectra was obtained on a JASCO V-530 UV/VIS spectrophotometer. NMR spectra were run on Varian Unity-plus 400 MHz FT-NMR, Varian Mercury-plus 400 MHz FT-NMR and Varian Unity-plus 600 MHz FT-NMR. The chemical shift (*δ*) values are in ppm (part per million) with DMSO and CD_3_OD as internal standard, and coupling constants (*J*) are in Hz. Low resolution ESI-MS spectra were obtained on an API 3000TM (Applied Biosystems) in positive or negative mode (solvent: CH_3_OH), high resolution ESI-MS spectra were obtained on a Bruker Daltonics APEX II 30e spectrometer in positive or negative mode (solvent: CH_3_OH). Circular dichroism was measured on a Jasco J-810 spectrophotometer. Shimadzu LC-10AT pump, Shimadzu SPO-M10A diode array detector, Shimadzu SIL-10A autoinjector and Varian Polaris 5 C18-A (250×4.6mm) were employed for the HPLC qualitative and quantitative analysis. Jasco PU-980 pump, Jasco UV-970 detector and Discovery HS C18 column (250×10 mm) were employed for separations. Silica gel 60 (70-230, 230-400 mesh, Merck), Sephadex LH-20 and Diaion HP-20 were used for column chromatography (CC). Silica gel plates (Kieselgel 60, F254, 0.20nm, Merck) were used for TLC.

### Plant Materials

The immature seeds (2.28 Kg), mature seeds (2.3 Kg), stems (1.21 Kg), leaves (0.2 Kg) and roots (0.15 Kg) of *N. foetida*, were collected in 2004 from the farm of Taiwan Sugar Corporation, Tainan, Taiwan. Only immature seeds were used to isolate pure constituents.

### Extraction and Isolation

The shade-dried immature seeds (2.28 Kg) were ground and extracted five times with MeOH (4 L) at room temperature. The methanolic extract (91.61 g) was partitioned between CHCl_3_ and *n*-BuOH with water. The CHCl_3_ fraction (2 g) was subjected to CC eluting with gradient mixtures of CHCl_3_-MeOH to afford nine fractions (Fr. C1**-**C9). The precipitate (181.61 mg) of Fr. 5 was filtered and rechromatographed with gradient mixtures of CHCl_3_-MeOH to afford several subfractions. The camptothecinoids were obtained from subfractions 4-6 and were further purified by reverse phase HPLC with acetonitrile/water (25/75) to afford compounds **1** (1.69 mg), **3** (11.51 mg), **4** (3.79 mg), **5a**/**5b** (8.19 mg) and **6a**/**6b** (8.12 mg) [17]. Compounds **7** (3.34 mg) and **8** (2.14 mg) were purified from subfractions 7-8 in a similar way.

The *n*-BuOH extract (6.80 g) was separated by Diaion HP-20 CC and eluted with a stepwise gradient of water-MeOH (pure water, water/MeOH 75/25, water/MeOH 50/50, water/MeOH 25/75, pure MeOH and pure aceton) and originated six fractions (Fr. B1-B6). Among them, Fr. B4 was separated by Sephadex LH-20 gel, followed by purification by reverse phase HPLC to afford compounds **2a**/**2b** (2.07 mg), **10** (3.03 mg) [19] and **11** (6.04 mg) [20].

### Compound characterization

*9-Methoxy-18,19-dehydrocamptothecin* (**1**). Pale yellow amorphous powder (1.69 mg); 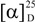 +24.5° (CHCl_3_; *c* 0.20); UV 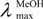 nm (log ε): 261 (3.66), 357 (3.49); IR (neat) *ν*_max_ 3406, 2922, 1745, 1652, 1600, 1450, 1372, 1118 cm^-1^; ^1^H- and ^13^C-NMR (DMSO), see Table 1; HRESIMS *m/z* 377.1135 [M+H]^+^, (calcd. for C_21_H_17_N_2_O_5_, 377.1137).

*5-Hydroxy-mappicine-20-O-β-glucopyranoside* (**2a**/**2b**). White amorphous powder (2.07 mg); 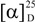 -39.2° (CHCl_3_; *c*0.20); UV 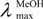 nm (log ε): 223 (3.96), 255 (3.82), 357 (3.63); IR (neat) *ν*_max_ 3397, 2929, 1670, 1596, 1457, 1340, 1195, 1113 cm^-1^; ^1^H- and ^13^C-NMR (CD_3_OD) see Table 1; HRESIMS *m/z* 485.1925 [M+H]^+^, (calcd. for C_25_H_29_N_2_O_8_, 485.1924).

### Crude samples prepared from different parts of N. foetida for qualitative and quantitative analysis

Dried leaves, stems, roots, mature seeds, and immature seeds were ground, 10 g were weighed and extracted with methanol at 24-25°C for 5 days, to mimic the large scale extraction conditions. All extracts were evaporated under reduced pressure to give five residues (leaves: 3.05 g, stems: 0.93 g, roots: 1.60 g, mature seeds: 0.44 g and immature seeds: 0.42 g). Each dry extract (5.0 mg) was dissolved in DMSO/MeCN (0.6 mL), filtered on a pre-column and injected to HPLC (each injection was 30 µL).

### Preparation of reference samples

Camptothecinoids were isolated from immature seeds of *N. foetida* and their structures were determined by spectroscopic methods. These reference compounds were used for qualitative analysis. Standard stock solutions containing 1 mg/mL of compounds **3, 4** were prepared with 1 mg compounds **3**, **4** in 1.0 mL DMSO for quantitative analysis. Standard sample solutions were injected (injection volume: 10 µL) directly into the HPLC system.

### Analytical HPLC

HPLC analyses were performed on a Shimadzu model LC-10AT HPLC (Japan) equipped with a two solvent delivery system, a SIL-10A automatic sample injector and a model SPD-M10A diode array detector. The detector was at 365 nm. Data acquisition and quantification were performed using Shimadzu Class-VP software (version: 6.12SP5). Chromatography was carried out on a Varian Polaris 5 C18-A (250×4.6mm i.d.) column. Isocratic elution was performed with water and HPLC-grade acetonitrile/H_2_O (25:75, v/v) at a flow rate of 1 mL/min. The solvents were filtered through a 0.45 μm filter prior to being used. Total HPLC running time for the assay was 22 minutes.

### Calibration

In the standard HPLC chromatogram, six different concentrations of compounds **3** and **4**, in the linear range of 0.015 to 0.5 mg/mL, were prepared in DMSO, respectively. Six replicates (n=6) of each concentration were subjected to HPLC.

### Cytotoxicity assay

Fractions and isolates were tested against lung (A549), breast (MEA-MB-231 and MCF7), and liver (HepG2 and Hep3B) cancer cell lines using established colorimetric MTT assay protocols [21]. Doxorubicin was used as a positive control. Freshly trypsinized cell suspensions were seeded in 96-well microtiter plates at densities of 5000-10000 cells per well with tested compounds added from DMSO stock solution. After 3 days in culture, attached cells were incubated with MTT (0.5 mg/mL, 2 h) and subsequently solubilized in DMSO. The absorbance was measured at 550 nm using a microplate reader. The IC_50_ is the concentration of agent that reduced cell growth by 50% under the experimental conditions.
